# Exploring deep learning radiomics for classifying osteoporotic vertebral fractures in X-ray images

**DOI:** 10.3389/fendo.2024.1370838

**Published:** 2024-03-28

**Authors:** Jun Zhang, Liang Xia, Jiayi Liu, Xiaoying Niu, Jun Tang, Jianguo Xia, Yongkang Liu, Weixiao Zhang, Zhipeng Liang, Xueli Zhang, Guangyu Tang, Lin Zhang

**Affiliations:** ^1^ Department of Radiology, Shanghai Tenth People’s Hospital, Clinical Medical College of Nanjing Medical University, Shanghai, China; ^2^ Department of Radiology, Sir RunRun Hospital, Nanjing Medical University, Nanjing, China; ^3^ Department of Neonates, Dongfeng General Hospital of National Medicine, Hubei University of Medicine, Shiyan, China; ^4^ Department of Radiology, The Affiliated Taizhou People’s Hospital of Nanjing Medical University, Taizhou, China; ^5^ Department of Radiology, Jiangsu Provincial Hospital of Traditional Chinese Medicine, Nanjing University of Chinese Medicine, Nanjing, China; ^6^ Department of Radiology, Shanghai Tenth People’s Hospital, Tongji University School of Medicine, Shanghai, China

**Keywords:** osteoporotic vertebral fractures, classification, X-ray computed tomography, deep learning, radiomics

## Abstract

**Purpose:**

To develop and validate a deep learning radiomics (DLR) model that uses X-ray images to predict the classification of osteoporotic vertebral fractures (OVFs).

**Material and methods:**

The study encompassed a cohort of 942 patients, involving examinations of 1076 vertebrae through X-ray, CT, and MRI across three distinct hospitals. The OVFs were categorized as class 0, 1, or 2 based on the Assessment System of Thoracolumbar Osteoporotic Fracture. The dataset was divided randomly into four distinct subsets: a training set comprising 712 samples, an internal validation set with 178 samples, an external validation set containing 111 samples, and a prospective validation set consisting of 75 samples. The ResNet-50 architectural model was used to implement deep transfer learning (DTL), undergoing -pre-training separately on the RadImageNet and ImageNet datasets. Features from DTL and radiomics were extracted and integrated using X-ray images. The optimal fusion feature model was identified through least absolute shrinkage and selection operator logistic regression. Evaluation of the predictive capabilities for OVFs classification involved eight machine learning models, assessed through receiver operating characteristic curves employing the “One-vs-Rest” strategy. The Delong test was applied to compare the predictive performance of the superior RadImageNet model against the ImageNet model.

**Results:**

Following pre-training separately on RadImageNet and ImageNet datasets, feature selection and fusion yielded 17 and 12 fusion features, respectively. Logistic regression emerged as the optimal machine learning algorithm for both DLR models. Across the training set, internal validation set, external validation set, and prospective validation set, the macro-average Area Under the Curve (AUC) based on the RadImageNet dataset surpassed those based on the ImageNet dataset, with statistically significant differences observed (P<0.05). Utilizing the binary “One-vs-Rest” strategy, the model based on the RadImageNet dataset demonstrated superior efficacy in predicting Class 0, achieving an AUC of 0.969 and accuracy of 0.863. Predicting Class 1 yielded an AUC of 0.945 and accuracy of 0.875, while for Class 2, the AUC and accuracy were 0.809 and 0.692, respectively.

**Conclusion:**

The DLR model, based on the RadImageNet dataset, outperformed the ImageNet model in predicting the classification of OVFs, with generalizability confirmed in the prospective validation set.

## Introduction

Osteoporosis (OP) is a prevalent metabolic bone disease, characterized by diminished bone strength and an elevated risk of fractures (1). Among osteoporotic fractures (OFs), those occurring in the spine, termed osteoporotic vertebral fractures (OVFs), are the most common (2). OVFs are fractures resulting from low-energy trauma, akin to a fall from a standing position in adults, and are linked to substantial rates of disability and mortality (3). Postmenopausal women exhibit an approximate 40% prevalence of OVFs, while elderly men experience rates between 25% and 33%. In China, a new case of OVF arises approximately every 17.4 seconds (4).

OVFs often manifest subtly with a complex clinical presentation. Acknowledged by both national and international scientific communities, the consensus is that achieving a comprehensive and precise classification of OVFs holds significant value for early diagnosis, treatment, and prognosis assessment (5). Several classification methods for OVFs have been put forth, such as the Genant semi-quantitative method (6), Heini classification (7), Osteoporotic Fracture Classification (8), and ASTLOF (9). However, none of these methods have garnered international acceptance (10). The ASTLOF classification, recognized for its good reproducibility and clinical utility (11), was selected as the standard for classification in this study. Conventional radiography, characterized by its speed, practicality, and cost-effectiveness, boasts the additional advantage of a relatively low radiation dose (0.3-0.7 mSv). The National Institute for Health and Care Excellence recommends conventional X-ray imaging as the preferred modality for OVFs (12). Given the widespread availability of digital radiography (DR) equipment in primary healthcare facilities across China, including remote areas (13), the development of a predictive model for OVFs based on X-ray images holds significant clinical importance.

Radiomics is a field focused on extracting numerous features from medical images, facilitating the evaluation of microstructural changes in trabecular bone (14), discerning osteoporosis (15), distinguishing between acute and chronic vertebral fractures (16), and forecasting the risk of vertebral fractures (17). Recent advancements in deep learning and radiomics have led to significant progress in the classification and evaluation of OVFs, as highlighted by several key studies. Dong et al. (6) used chest and lumbar spine X-ray images from the MrOS (The Osteoporotic Fractures in Men) dataset and classified OVFs into moderate/severe fractures and normal/minor fractures based on the Genant semi-quantitative method, utilizing GoogLeNet for training to subtype OVFs. The model achieved an AUC of 0.99, demonstrating high diagnostic performance in identifying moderate/severe OVFs. However, the MrOS study was limited to male OVFs patients from six clinical centers in the United States, necessitating further testing to determine the model’s applicability to females and international populations. Zhang et al. (18) employed U-net and U-Graph Convolution Network for thoracolumbar localization and classification, achieving AO classification through a multi-branch output network. The system’s accuracy was 97.93% for fracture detection and 79.56% for AO classification assessment, indicating its capability to accurately evaluate OVFs based on AO classification. However, this study only involved A1-4 type fractures, excluding Type B and C fractures. Dong et al. (19) trained models (GoogLeNet, Inception-ResNet-v2, EfficientNet-B1, and two ensemble algorithms) based on the m2ABQ classification method for OVFs, using ImageNet pre-trained models for transfer learning. The best-performing model achieved excellent results (AUCs of 0.948 for the local test set and 0.936 for the MrOS test set), yet the authors did not analyze misclassified cases nor explore how image features affect the output of each model.

DLR is a branch of machine learning, focuses on deriving profound image features, often using pretrained network architectures like ResNet50 on ImageNet. ImageNet, with its extensive collection of natural world images, plays a vital role in effective transfer learning, requiring a degree of resemblance between the model’s training imagery and the target application (20). On the other hand, RadImageNet, an open-source database of medical images derived from various medical sources, is posited to be more apt for DTL in comparison to ImageNet (21). Therefore, in this research, X-ray images of the thoracolumbar vertebrae from different medical centers were used to develop DTL-based predictive models for OVFs identification, pre-training separately on RadImageNet and ImageNet datasets and following the ASTLOF classification system. The efficacy of these models was then assessed and compared using data from multiple centers.

## Methods

### Design and participants

This study utilized X-Ray images from multicenter. Following thorough review and approval by the respective hospital ethics committees, the retrospective dataset was granted an exemption from the need for patient informed consent. In contrast, patients included in the prospective validation set were duly informed and provided written consent by signing informed consent forms.

To ensure the robustness and generalizability of our DLR model for classifying OVFs using X-ray images, we divided our dataset into four distinct subsets: training, internal validation, external validation, and prospective validation. The training set was used to develop the model, allowing it to learn to identify patterns and features indicative of OVFs.The internal validation set was employed to fine-tune the model parameters and mitigate overfitting, providing an initial assessment of the model’s performance. The external validation set was included to test the model’s generalizability to new, unseen data from different populations or settings, crucial for evaluating its applicability in diverse clinical environments. Finally, the prospective validation set was utilized to validate the model on prospectively collected data, offering insights into its real-world performance and ensuring its reliability and applicability over time and under varied conditions.

**Figure 1:**
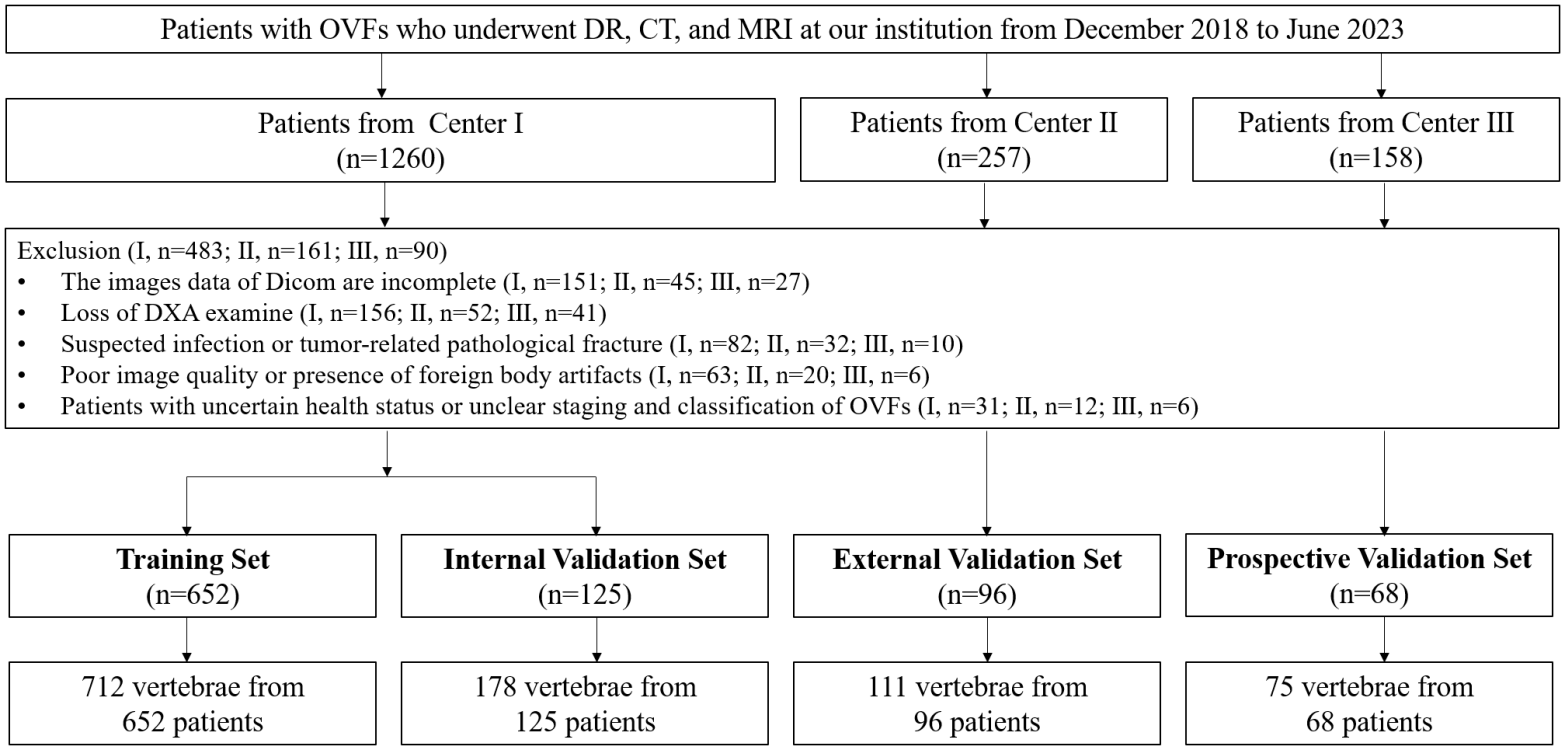
Flowchart summarizes patient selection and allocation to the training set, internal and external validation set and prospective validation set of the multicenter study.

**Figure 2:**
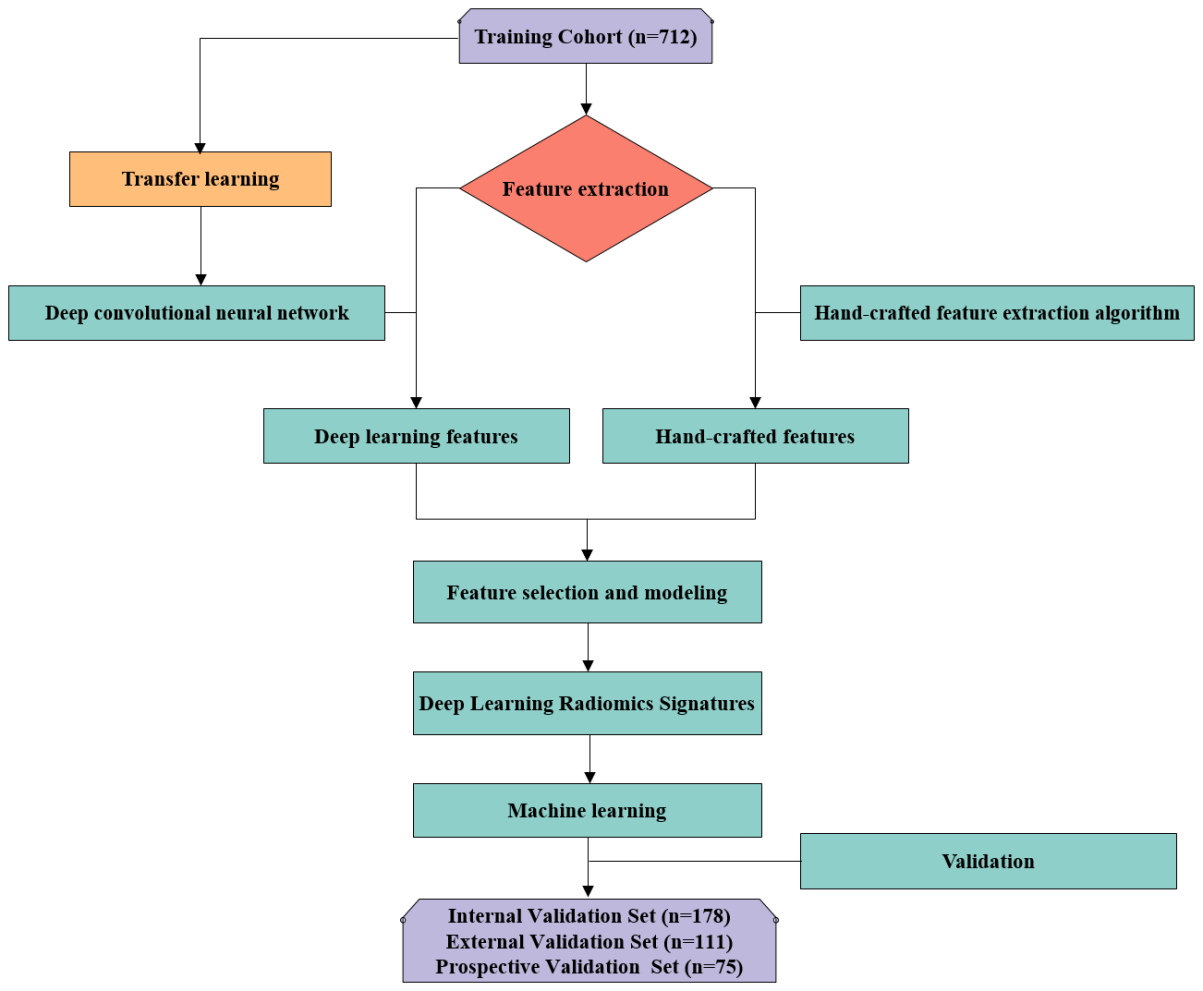
Flowchart in this study.

**Figure 3:**
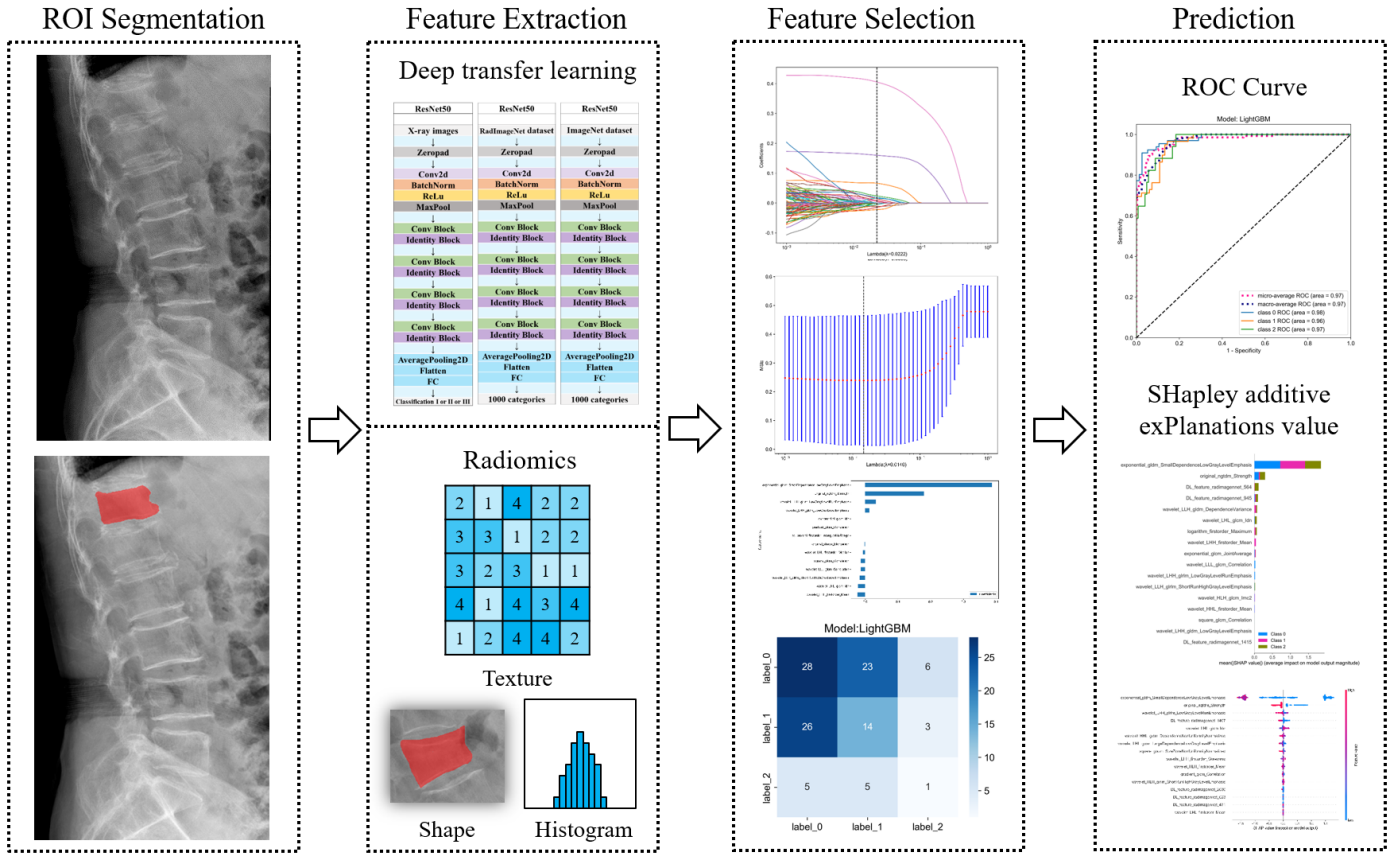
Workflow of deep learning radiomics workflow.

To form the training and validation sets (both internal and external), patient data from Center I and Center II, including X-ray, CT, and MRI related to OVFs, were gathered from December 2018 to December 2022. The selection criteria for the cases were defined as follows: Inclusion criteria included patients aged 50 years or older diagnosed with OVFs (22), encompassing those without any history of trauma or with only minor trauma incidents; availability of complete Dicom datasets for X-ray, CT, and MRI examinations, conducted within a maximum two-week interval; and comprehensive clinical data availability, including gender, age, and results from Dual-energy X-ray absorptiometry (DXA) exams. The exclusion criteria were suspected fracture cases due to infection or tumors; poor image quality or presence of artifacts; and patients with unclear health status or fracture classification. From January 2023 to June 2023, an independent prospective validation set was added from Center III, following the same inclusion and exclusion criteria. **Figure 1** provides a detailed schematic of the case selection process, illustrating the random assignment of cases to the training set and internal validation set in an 8:2 ratio. For further information about the case collection process, grouping, image preprocessing, feature extraction and analysis, and model development, refer to the flowcharts and DLR workflow in **Figures 2** and **3**.

### Classification

All cases were classified and graded using the ASTLOF, which assigns scores to OVFs based on four aspects: vertebral morphology, MRI imaging, bone density values, and clinical symptoms (23). The descriptions are as follows: ① Morphological changes (CT scan): Normal = 0 points, compression = 1 point, burst = 2 points; ② MRI examination: Normal = 0 points, high signal changes on fat-suppressed imaging = 1 point, presence of vacuum phenomenon or fluid sign within the vertebral body = 2 points; ③ Bone density: T-score > -2.5 = 0 points, -3.5 ≤ T-score ≤ -2.5 = 1 point, T-score ≤ -3.5 = 2 points; ④ Clinical presentation: No significant pain = 0 points, position-induced lumbago = 1 point, persistent pain or presence of neurological symptoms = 2 points. Based on the total score, the classification is as follows: Class 0 (total score ≤ 3 points, conservative treatment), Class 1 (total score = 4, conservative or surgical treatment), Class 2 (total score ≥ 5 points, surgical treatment). Two radiologists, A and B, with 6 and 10 years of experience, respectively, evaluated the classification of OVFs. In instances of conflicting outcomes, a conclusive decision was reached through collaborative consultation.

### Acquisition of X-ray images and analysis of clinical baseline features

Data on age, gender, and T- score from DXA were retrieved from the clinical health records system. Details about the imaging devices used for X-ray, CT, and MRI, along with the associated imaging parameters, are provided in **Supplementary Table S1**. In the process of X-ray imaging for the thoracic or lumbar spine, it was essential to align the X-ray beam’s central ray perpendicular to either the 7th thoracic vertebra or the 3rd lumbar vertebra. The analyses and processing in this research were conducted using the lateral view images acquired from vertebral DR examinations.

### Image segmentation

Precision in segmenting vertebral bodies is crucial for the analysis that follows. In our research, physicians carried out the segmentation manually. Initially, physician A imported the X-ray images into the ITK-SNAP software (version 3.8.0, available at http://www.itksnap.org). During this process, the edges of the fractured vertebral bodies were carefully outlined and filled by hand. Care was taken to exclude adjacent intervertebral discs, pedicles, and any surrounding tissue

**Figure 4:**
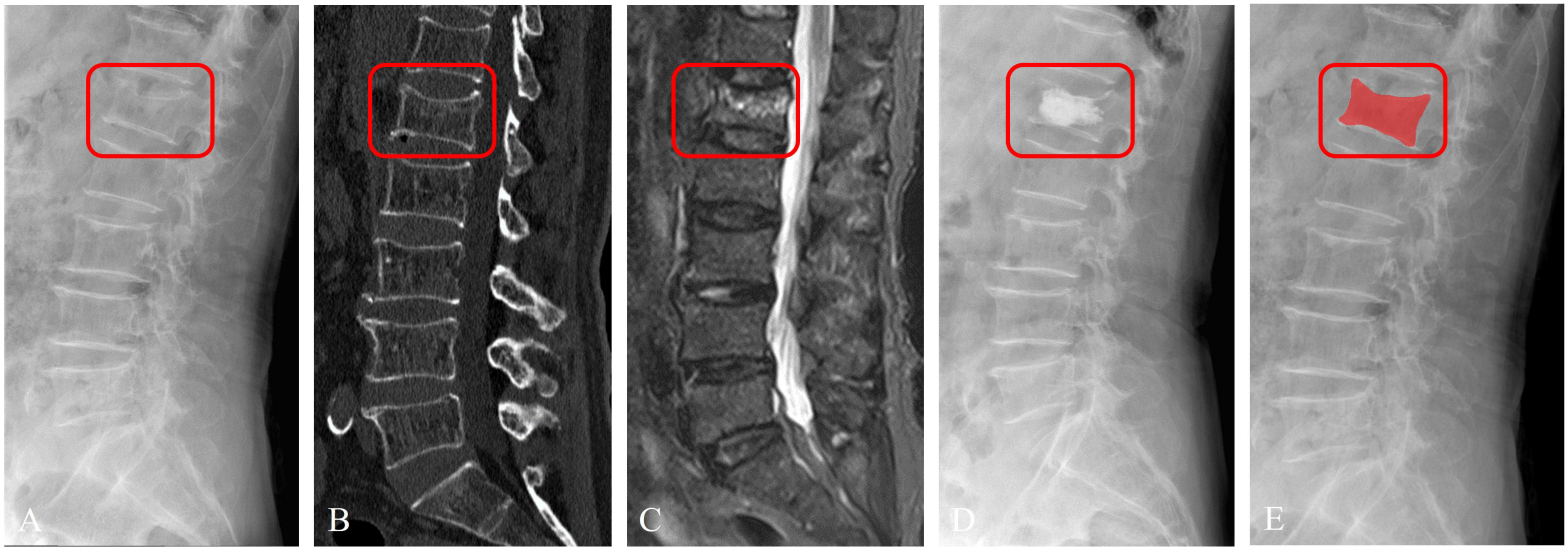
Imaging segmentation of the vertebral body in a 63-year-old female patient diagnosed with acute OVFs, rated an ASTLOF score of 4. The set includes: **(A)** lateral views on X-ray image, **(B)** sagittal spine CT image without contrast enhancement, **(C)** sagittal T2-weighted, fat-suppressed MR image displaying hyperintensity indicative of acute OVFs, **(D)** lateral X-ray image post percutaneous vertebral augmentation, and **(E)** ROI identified on lateral X-ray image.

The outlining of the region encompassing the fractured vertebral body was carefully executed to ensure completeness and accuracy before being saved as a mask file in ‘nii’ format (refer to **Figure 4**). A month subsequent to this, 30 patients were randomly chosen from the training dataset, and their images were re-delineated by both physician A and physician B. To assess the consistency of vertebral body delineation, both intra-observer and inter-observer reliability were measured using the intraclass correlation coefficient (ICC) among the participants.

### Radiomics and DTL features extraction

**Figure 5:**
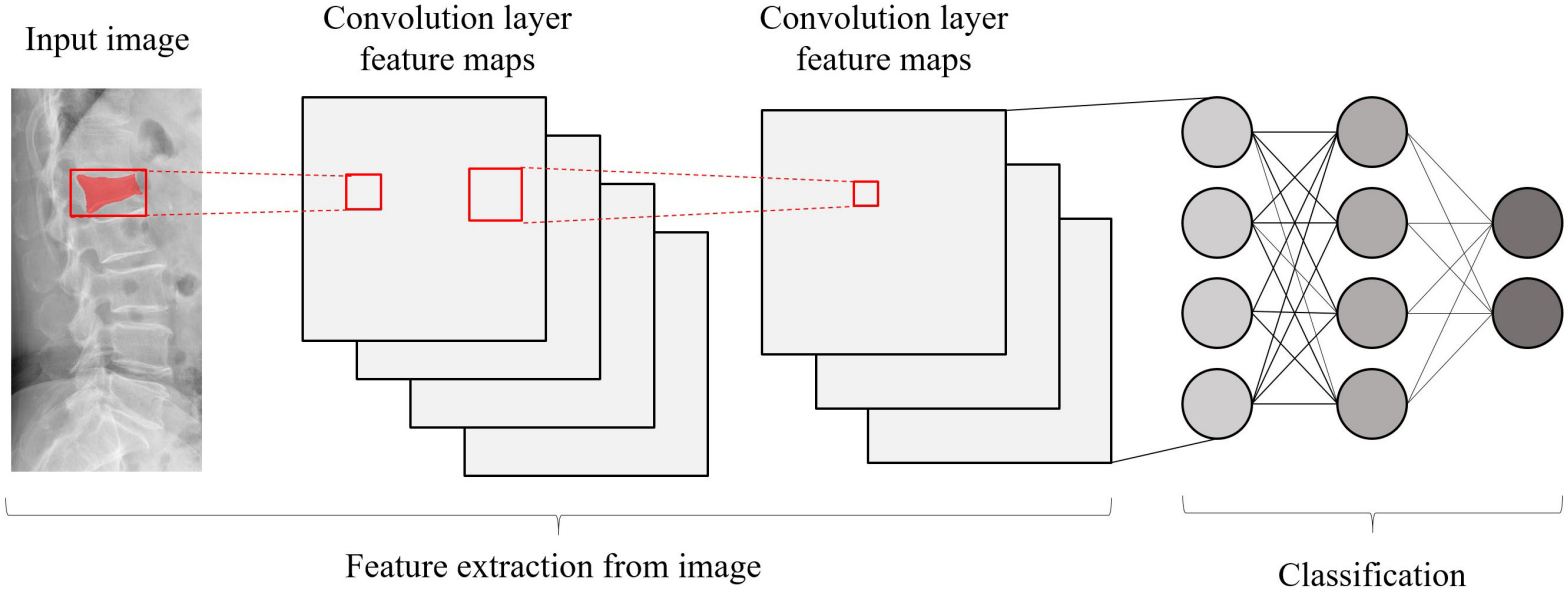
A basic architecture of a convolutional neural network.

**Figure 6:**
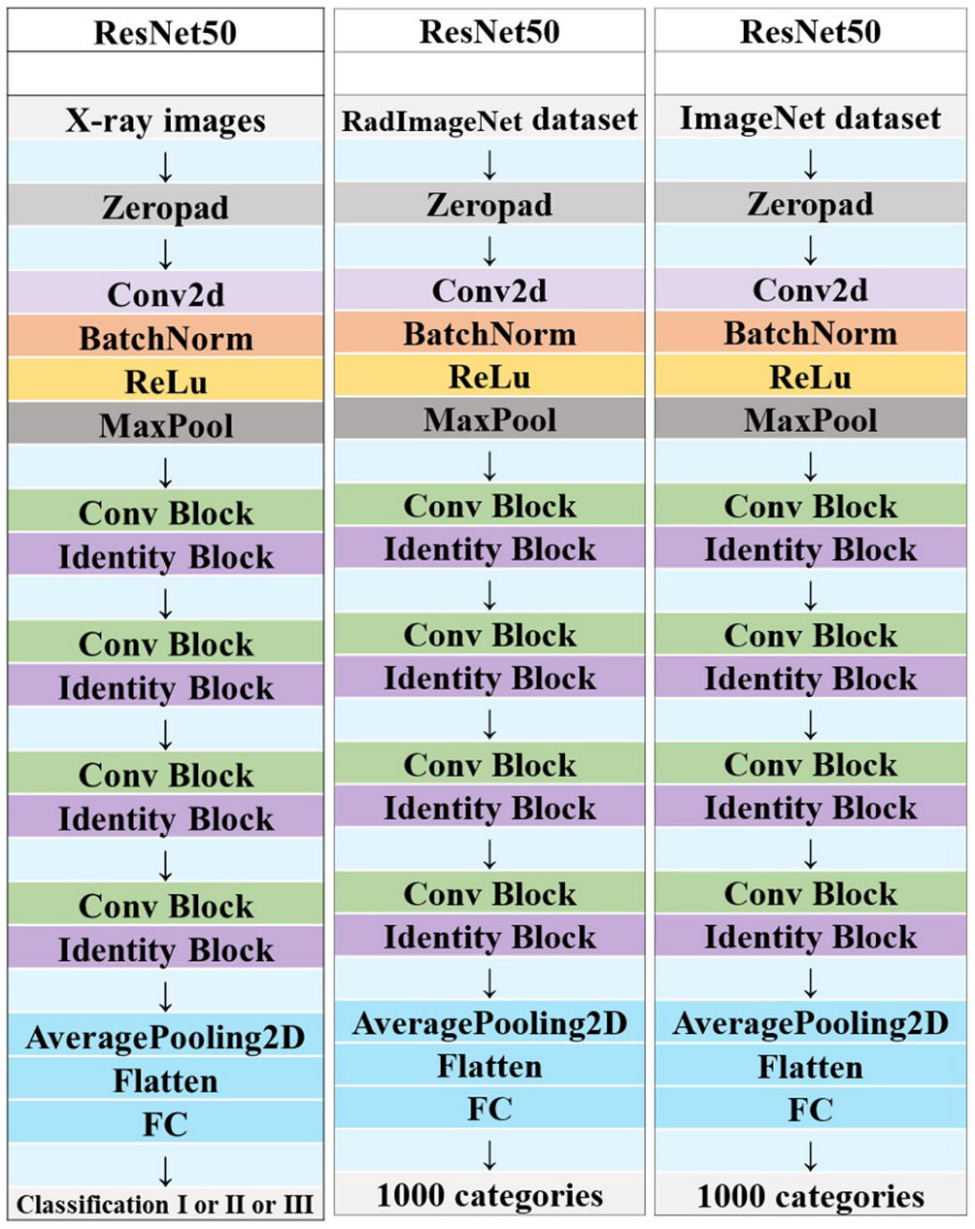
Schematic diagram of the deep convolutional neural network pre-training and fine-tuning network structure.

Each image underwent Z-score normalization to minimize variations across the images. The protocol for feature extraction adhered to the standards set by the Image Biomarker Standardization Initiative, as referenced in (24). Subsequently, the extracted radiomics features were harmonized across different centers utilizing the Combat method, detailed in (25), to mitigate discrepancies in the data. Utilizing the PyTorch deep learning framework within the Python 3.6 environment, the DTL method was executed, aligning with methodologies used in prior research (26). For this study, the ResNet50 model (**Figures 5**, **6**) was selected as the foundational model for DTL, with a meticulously adjusted learning rate to enhance performance. Since the transfer features were selected from the second-to-last layer of the model (Average-Pooling layer), we divided the model parameters into two parts: the Backbone part and the Task-specific part. The initialization of the Backbone part used the pre-trained model parameters from RadImageNet (27) and ImageNet. The task-specific segment of the model received a random parameter initialization, consistent with the cosine annealing approach to learning rate decay (28). In our study, we employed the following hyperparameters for training our model: optimizer set to ‘Adam’, a batch size of 8, and training for 30 epochs. This learning rate was dynamically adjusted based on iteration count, as detailed in **Supplementary S2**. For more details, please check the **Supplementary S3**.

### Feature selection and fusion

Initially, the selection of radiomic features was based on both their repeatability and minimal redundancy, focusing on those with an intraclass correlation coefficient (ICC) of 0.8 or higher (29). For features demonstrating high repeatability, Spearman’s rank correlation coefficient was employed to evaluate the inter-feature correlations. In instances where the correlation between any two features exceeded 0.9, only one of these features was preserved. To ensure maximum representation of the features, a strategy of greedy recursive elimination was applied. This involved systematically removing the feature with the greatest redundancy at each step. In the final step, the LASSO algorithm was employed. This method shrinks regression coefficients to zero by building a penalty function denoted as λ. Through this process, stable radiomics features were selected for inclusion in the LASSO-Cox analysis. For more details about the process of feature selection and fusion, please refer to **Supplementary S4**.

### Development of the deep learning radiomics model

To prevent data leakage, model training was exclusively conducted on the training dataset. Subsequent to the selection and integration of features, various machine learning classifiers were developed using the scikit-learn library. These classifiers encompassed a range of algorithms, including Logistic Regression (LR), Extremely Randomized Trees (Extratrees), Support Vector Machine (SVM), Light Gradient Boosting Machine (LightGBM), Multilayer Perceptron (MLP), Random Forests (RF), eXtreme Gradient Boosting (XGBoost) and k-Nearest Neighbor (KNN).

To optimize model training on the training dataset, the grid search algorithm was implemented, allowing for the adjustment of commonly used parameters within each model. A comparative evaluation of the performance across various classification models was conducted. To determine the most effective parameters for these models, 5-fold cross-validation was employed, leading to the identification of optimal fused feature labels. Furthermore, the significance of different features was assessed using the SHapley Additive exPlanations (SHAP) value (accessible at https://github.com/slundberg/shap). This method quantifies the contribution of each feature to the predictive outcomes, thereby elucidating their importance.

### Model evaluation and statistical analysis

In the realm of machine learning, a fundamental method for handling multi-class classification tasks involves the concept of decomposition. This approach entails dividing the multi-class problem into multiple binary classification tasks. In our study, we employed the widely recognized ‘One-vs-Rest’ strategy for such multi-class scenarios. As an example, under this strategy, if Class 0 is designated as positive, then Class 1 and Class 2 are considered negative. Similarly, when Class 1 or Class 2 is assigned the positive label, the other two categories are automatically labeled as negative. Consequently, a trio of ‘One-vs-Rest’ (OvR) classification models were developed and trained. To assess the efficacy of these prediction models, Receiver Operating Characteristic (ROC) curves were constructed, and various metrics including the Area under the Curve (AUC), accuracy, sensitivity, and specificity were calculated. The models’ capability to generalize was then appraised using internal and external validation sets, along with a prospective validation set. For a comprehensive evaluation of the multi-class task, this study utilized both macro-averaged and micro-averaged AUC as the metrics of choice, which are different approaches to aggregate multiple confusion matrices. The macro-averaged AUC approach involves computing the AUC for each individual class and then averaging these AUCs across all classes. On the other hand, the micro-averaged AUC method amalgamates the predictive outcomes of all classes into a singular confusion matrix, from which the collective AUC is derived. Statistical analyses in this study were conducted using the R software (version 4.0.3). To conclude, the Delong test was applied to assess the AUC differences between prediction models, with a P-value less than 0.05 deemed indicative of a statistically significant variance.

## Result

### Clinical baseline characteristics

In our study, 942 patients who satisfied the inclusion criteria were enrolled, ranging in age from 50 to 97 years, with an average age of 69.34 ± 10.19 years. The cohort consisted of 678 female and 264 male patients. Based on the DXA T-score classification, the study included 16 patients with normal bone mass, 205 with reduced bone mass, and 721 with osteoporosis. Within this group, 86 patients had experienced 2 osteoporotic vertebral fractures (OVFs), and 24 had 3 OVFs, bringing the total number of vertebral fractures analyzed to 1076. The demographic and clinical characteristics of these patients are detailed in **Table 1**, while **Table 2** outlines their treatment categorization across three different classes. In Class 0 (1-3 points), 357 patients (72.9%) underwent conservative treatment, and 113 patients (27.1%) underwent surgery. In Class 1 (4 points), conservative treatment was administered to 159 patients (34.5%), with the remaining 302 patients (65.5%) receiving surgical treatment. Lastly, in Class 2 (5-8 points), conservative treatment was given to 37 patients (29.6%), and surgical treatment was opted for in 85 patients (70.4%).

**Table 1:** Baseline Characteristic of patients with OVFs in the Training set, Internal/External validation set and Prospective validation set.

Characteristic	Training set (n=712)	Interval Validation set (n=178)	External Validation set (n=111)	Prospective validation set (n=75)
Sex, No. (%)				
Female	531 (74.6)	123 (69.1)	70 (63.1)	51 (68.0)
Male	181 (25.4)	55 (30.9)	41 (36.9)	24 (32.0)
Age (years)				
Mean (range)	70.38±10.49	68.01±10.73	65.54±9.19	65.83±11.32
DXA T-score				
Mean (range)	-2.7±0.70	-3.0±0.81	-2.7±0.53	-2.9±0.69
Fracture location, No. (%)				
Thoracic	205 (28.8)	47 (26.4)	19 (17.1)	19 (25.3)
Lumbar	507 (71.2)	131 (73.6)	92 (82.9)	56 (74.7)
Fracture staging, No. (%)				
Acute	472 (66.3)	104 (58.4)	76 (68.5)	53 (70.7)
Chronic	240 (33.7)	74 (41.6)	35 (31.5)	22 (29.3)
ASTLOF score, No. (%)				
1-3 points	330 (46.3)	75 (42.1)	53 (47.7)	32 (42.7)
4 points	294 (41.3)	85 (47.8)	49 (44.1)	33 (44.0)
5-8 points	88 (12.4)	18 (10.1)	9 (8.2)	10 (13.3)
Therapeutic method, No. (%)				
Conservative treatment	354 (49.7)	97 (54.5)	60 (54.1)	42 (56.0)
PVA	290 (40.7)	58 (32.6)	36 (32.4)	19 (25.3)
Open surgery	68 (9.6)	23 (12.9)	15 (13.5)	14 (18.7)

OVFs, osteoporotic vertebral fractures; DXA, Dual-energy X-ray absorptiometry; ASTLOF, Assessment System of Thoracolumbar Osteoporotic Fracture; PVA, Percutaneous Vertebral Augmentation.

**Table 2:** OVFs according to the ASTLOF classification and their distribution among the therapeutic method.

Classification	Conservative treatment (n=553, %)	PVA (n=403, %)	Open surgery (n=120, %)
Class 0 (1-3points)	357 (64.6)	98 (24.3)	35 (29.2)
Class 1 (4points)	159 (28.7)	232 (57.6)	70 (58.3)
Class 2 (5-8points)	37 (6.7)	73 (18.1)	15 (12.5)

OVFs, osteoporotic vertebral fractures; ASTLOF, Assessment System of Thoracolumbar Osteoporotic Fracture; PVA, Percutaneous Vertebral Augmentation.

### Features selection (RadImageNet-based)

In the analysis, the LASSO-Cox regression model was applied to reduce the dimensionality of the combined features. The process of selecting the optimal penalty coefficient (λ set at 0.0222) and the feature selection methodology are illustrated in **Supplementary Figure S5**. This figure depicts how the coefficients of the features vary with changes in λ. Post the final round of feature selection, a total of 14 radiomics features and 3 DTL features were selected to form the composite features. Utilizing these features and their respective regression coefficients, the DTL_Radscore was formulated, as shown in **Supplementary Figure S6**. The detailed equation used to calculate the DTL_Radscore is available in **Supplementary S7**.

### Features selection (ImageNet-based)

In the application of the LASSO-Cox regression analysis, the selected penalty coefficient (λ) was determined to be 0.0126. The procedure used for selecting features, along with a curve graph that illustrates how the coefficients of the features change with λ, is presented in **Supplementary Figure S8**. Upon completion of the final feature selection process, a combination of 6 radiomics features and 6 deep transfer learning (DTL) features were maintained as the fused features. These features, along with their associated regression coefficients, were utilized to develop the DTL_Radscore, as depicted in **Supplementary Figure S9**. For a comprehensive understanding of the DTL_Radscore calculation, the formula is provided in **Supplementary S10**.

### Model construction and validation

**Figure 7:**
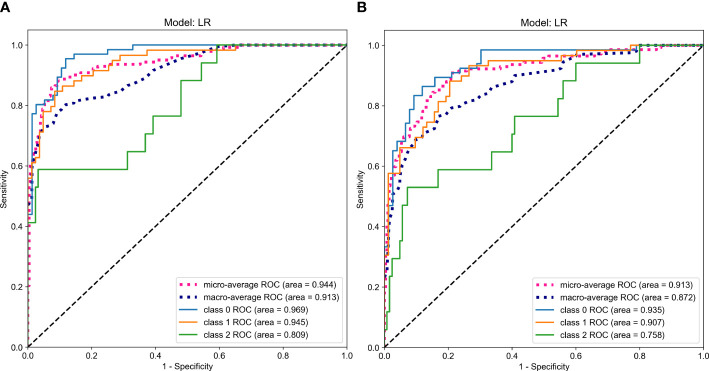
ROC Curves Comparing the Predictive Performance in the prospective validation set (**A**. RadImageNet, **B.** ImageNet).

**Figure 8:**
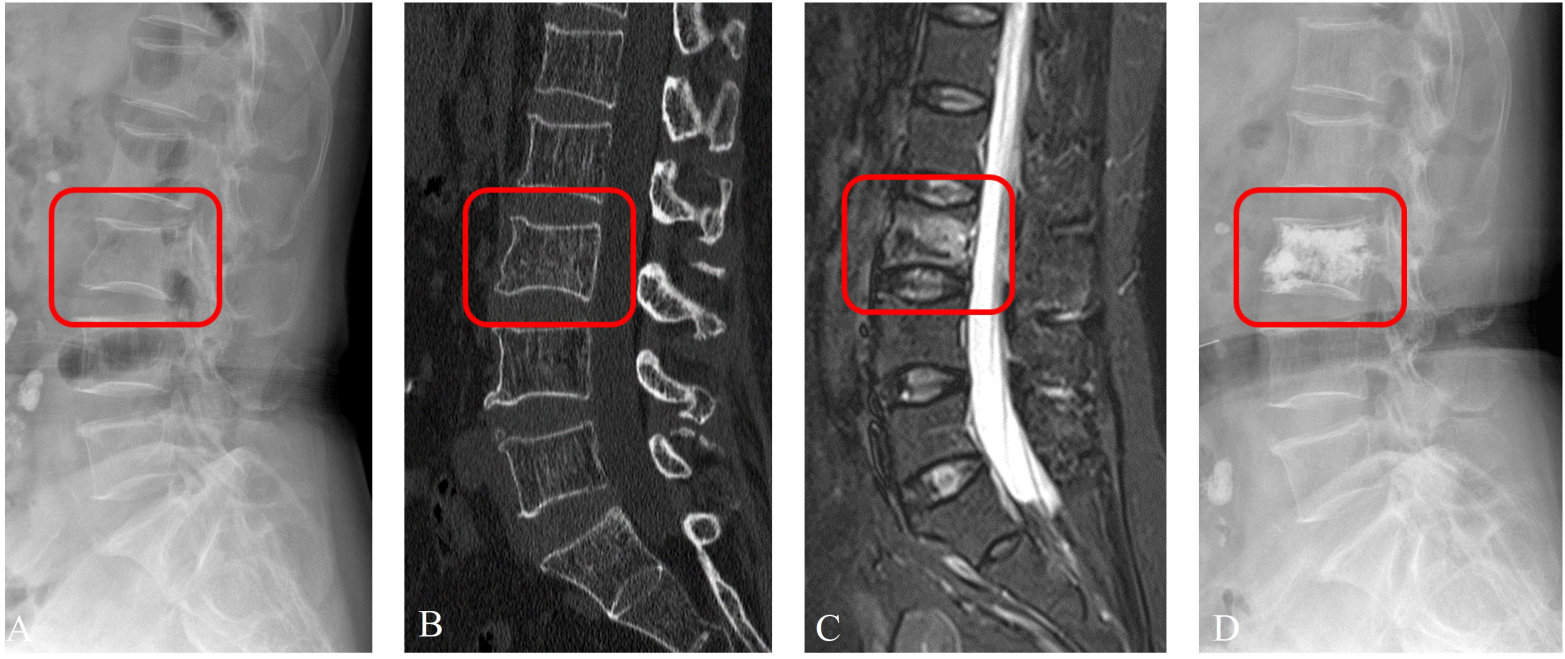
Prediction example based on RadImageNet model. An examples of a 65-year-old female patient with OVFs (ASTLOF score: 5) in the prospective validation set. This case was misclassified by the ImageNet model, but correctly reclassified as the Class 1 by the RadImageNet model (**A**. X-ray, **B**. CT, **C**. MRI, **D**. X-ray with PVA).

According to the comparison of macro-averaged AUC, accuracy, and F1-score, the LR algorithm performed the best in the fused feature models trained on RadImageNet (**Supplementary Table S11**) and ImageNet datasets (**Supplementary Table S12**). The validation results of the two sets of fused feature prediction models for the three-class classification task can be found in **Table 3**. Based on the RadImageNet dataset, the macro-averaged AUC for the training set, internal validation set, external validation set, and prospective validation set were all higher compared to the ImageNet dataset (0.913 vs 0.831, 0.926 vs 0.826, 0.940 vs 0.844, 0.913 vs 0.872). The Delong test revealed statistically significant differences (*P*<0.05) in all pairwise comparisons. **Figure 7** displays the ROC curves of the two sets of fused feature models in predicting OVF classifications in the prospective validation set. Based on the binary “OvR” strategy, the RadImageNet dataset model showed the most effective prediction for Class 0, with an AUC of 0.969 and accuracy of 0.863. The AUC and accuracy for predicting Class 1 were 0.945 and 0.875, respectively, while for predicting Class 2, they were 0.809 and 0.692, respectively. **Figure 8** shows cases where the prediction model based on the ImageNet dataset made classification errors, while the model based on the RadImageNet dataset made correct classifications.

**Table 3:** The classification performance of the models in the Training set, Internal/External validation set and Prospective validation set.

Model	Training set		Interval validation set		External validation set		Prospective validation set	
	Accuracy	AUC^#^	Accuracy	AUC	Accuracy	AUC	Accuracy	AUC
RadImageNet-based								
Class 0	0.846	0.959 (0.942-0.976)	0.835	0.958 (0.926-0.991)	0.827	0.981 (0.954-0.999)	0.863	0.969 (0.939-0.999)
Class 1	0.801	0.923 (0.899-0.948)	0.837	0.913 (0.868-0.958)	0.788	0.921 (0.865-0.977)	0.875	0.945 (0.903-0.988)
Class 2	0.813	0.852 (0.795-0.910)	0.857	0.897 (0.799-0.995)	0.444	0.904 (0.776-0.999)	0.692	0.809 (0.681-0.937)
Three classification^*^	0.825	0.913 (0.886-0.940)	0.837	0.926 (0.885-0.968)	0.802	0.940 (0.890-0.989)	0.852	0.913 (0.860-0.967)
ImageNet-based								
Class 0	0.731	0.894 (0.866-0.921)	0.782	0.939 (0.899-0.978)	0.860	0.895 (0.832-0.958)	0.769	0.935 (0.891-0.979)
Class 1	0.792	0.873 (0.842-0.905)	0.800	0.819 (0.756-0.882)	0.714	0.762 (0671-0.854)	0.754	0.907 (0.853-0.961)
Class 2	0.636	0.720 (0.650-0.790)	0.612	0.698 (0.557-0.838)	0.400	0.857 (0.708-0.999)	0.667	0.758 (0.619-0.896)
Three classification	0.752	0.831 (0.794-0.867)	0.787	0.826 (0.762-0.890)	0.766	0.844 (0.766-0.922)	0.761	0.872 (0.808-0.937)

^*^Date are macro-average, ^#^Date in parentheses are 95% confidence intervals.

AUC, Area under curve.

### Feature importance for LR multiclass classification models based on RadImageNet

To evaluate the contribution of each feature to the model predictions, the SHAP values for each feature were calculated. **Supplementary Figure S13** displays the features ranked by their global SHAPley additive explanation values for the three-class classification and each individual class. This helps assess their contribution to the model predictions. The SHAP decision plots in **Supplementary Figure S14** provide an intuitive visualization of the workflow of the prediction model in predicting Class 0, Class 1, and Class 2 in the prospective validation set.

## Discussion

In contrast to clearly identifiable traumatic vertebral fractures, osteoporotic vertebral fractures (OVFs) are typically more subtle and often remain undetected. Inadequate treatment of OVFs can compromise spinal stability and balance, potentially resulting in severe neurological damage and an elevated risk of mortality (30). Accurate classification of OVFs is crucial not only for precise diagnosis but also for guiding appropriate clinical interventions. Establishing an extensive and detailed classification system for OVFs is immensely valuable for assessing fracture risk, selecting suitable treatments, and evaluating long-term prognoses (31). This study believes that an ideal classification system should have the following characteristics: ① It should incorporate multidimensional evaluation using X-ray, CT, and MRI imaging parameters. ② It should consider the patient’s clinical symptoms, such as back pain and neurological symptoms. ③ Each classification should have specific treatment approaches. ④ It should have high reliability and reproducibility. ⑤ It should allow for the assessment of severity and prognosis based on the classification. The ASTLOF classification system takes into account vertebral morphology, MRI images, bone mineral density T-scores, and clinical symptoms. It is a comprehensive and systematic evaluation index that assigns scores and helps select targeted treatment plans. It is easy to use in clinical practice and has significant clinical guidance value. Studies have shown that this classification system has high consistency and reproducibility and can effectively guide clinical treatment (23).

In the past few years, the rapid advancements in artificial intelligence have significantly influenced research in the classification of osteoporotic vertebral fractures (OVFs), with a primary focus on detection methods. Studies have demonstrated that approaches based on deep learning and radiomics are superior to traditional methods of visual analysis (6, 32). Despite this progress, most current studies on OVF classification and detection have confined their research to dividing data from a single center into training and validation sets for internal validation purposes. Yet, variations in image scanning techniques, post-processing reconstruction, scanning parameters, and differences among equipment from various manufacturers can lead to considerable discrepancies in radiomics findings (33). Additionally, single-center studies lack data heterogeneity and may result in varying degrees of overfitting. Multi-center studies provide diverse radiological data, and prediction models undergo independent external validation, which can better interpret the heterogeneity of OVFs and align with the development of precision medicine. The strength of this study lies in the use of chest and lumbar spine X-ray images from three hospitals, employing the ASTLOF classification system, conducting DTL separately based on the RadImageNet and ImageNet datasets, constructing a predictive model that combines radiomics and DTL features, evaluating its performance in predicting OVFs classification, and validating it with an independent prospective external set.

The ImageNet dataset contains millions of natural images and has been widely used to train various deep learning models, finding extensive applications in various medical scenarios. However, the ImageNet dataset was primarily designed for natural images, and medical images have their own characteristics and challenges, such as high noise and low contrast. The limitations in using the ImageNet dataset for texture representation in medical imaging are notable, primarily because the dataset lacks the intricate 2D and 3D structures typical of human anatomy. Consequently, DTL that relies on the ImageNet dataset might not be fully applicable to specific medical contexts. In contrast, the RadImageNet database, encompassing over 1.35 million grayscale medical images, includes a diverse array of anatomical structures like bones, muscles, and nerves. Research indicates that this database can significantly enhance DTL’s effectiveness in medical applications and exhibits superior generalization abilities in such contexts (34).

The results of this study also confirm that the predictive model based on the RadImageNet dataset outperforms the ImageNet model. In the case of imbalanced samples, the “One-vs-Rest” strategy is generally chosen for multi-classification tasks (35). The predictive model using the “OvR” strategy in this study demonstrates satisfactory classification ability, being most effective in identifying Class 0 and 1 classifications. However, in the prospective validation set, the AUC and accuracy for predicting Class 2 are slightly lower, which may be related to the smaller sample size of Class 2. Additionally, a possible explanation is that the inducible back pain (score 1) or persistent pain (score 2) in patients is related to many factors that cannot be directly measured or quantified by radiomics, such as the patient’s overall health status and pain threshold. In the context of multi-classification tasks, the SHAP value is frequently utilized to determine the significance of features. These values reveal whether each predictive variable positively or negatively influences the outcome (36). Notably, the Small Dependence Low Gray Level Emphasis (SDLGLE) feature has the highest correlation coefficient. A higher SDLGLE value suggests a more irregular texture (37). In cases of acute OVFs, common indicators such as disruption of the vertebral endplate, fractures within the trabecular bone, and uneven vertebral body density are primary contributors to this irregular texture. Furthermore, acute OVFs may exhibit elevated signal alterations in T2-weighted imaging with fat suppression sequences (noted as score 1) or display indications of vacuum phenomena and effusions within the vertebral body (noted as score 2). These manifestations also contribute to the irregularity of the texture. While the interpretability of features derived from current deep transfer learning models warrants additional investigation, this does not impede the identification and mapping of lesion-specific features through convolutional operations. These identified features can then be leveraged in the construction and classification of models.

Our study demonstrates that the fusion feature model has strong clinical value in distinguishing OVFs classifications. However, there are still some limitations that can be further explored and addressed in future work. Firstly, there is an imbalance in the sample sizes of the three classifications, such as a relatively small sample size for Class 2, which may result in misclassification as Class 0 or 1 and subsequently reduce the overall classification accuracy. In the future, increasing the sample size to overcome this imbalance is expected to achieve more convincing validation results. Secondly, considering that the vertebral body is inherently a three-dimensional structure, reliance solely on lateral images may not encapsulate all its features. Future studies should include anterior-posterior images to ensure a more thorough feature representation. Lastly, the interpretability of deep learning features extracted using the DLR method remains limited. Advancing research into the interpretability of radiomics features is crucial for augmenting the clinical utility of the DLR approach in practical settings.

## Conclusion

Our study combines deep learning features with radiomics features based on the RadImageNet dataset to construct a predictive model for distinguishing OVFs classifications. Compared to the ImageNet dataset, this model has good clinical utility in predicting OVFs classifications and guiding treatment planning.

## Data availability statement

The original contributions presented in the study are included in the article/**Supplementary Material**. Further inquiries can be directed to the corresponding author.

## Ethics statement

The studies involving humans were approved by Institutional Ethics Committee of the Sir RunRun Hospital affiliated to Nanjing Medical University. The studies were conducted in accordance with the local legislation and institutional requirements. The participants provided their written informed consent to participate in this study. Written informed consent was obtained from the individual(s) for the publication of any potentially identifiable images or data included in this article.

## Author contributions

JZ: Data curation, Formal analysis, Funding acquisition, Investigation, Methodology, Software, Validation, Writing – original draft, Writing – review & editing. LX: Data curation, Formal analysis, Funding acquisition, Investigation, Methodology, Resources, Supervision, Validation, Visualization, Writing – review & editing. JL: Conceptualization, Formal analysis, Investigation, Project administration, Supervision, Writing – review & editing. XN: Conceptualization, Formal analysis, Investigation, Methodology, Validation, Visualization, Writing – review & editing. JT: Data curation, Formal analysis, Project administration, Software, Writing – review & editing. JX: Software, Writing – review & editing. YL: Data curation, Writing – review & editing. WZ: Data curation, Writing – review & editing. ZL: Investigation, Supervision, Writing – review & editing. XZ: Data curation, Writing – review & editing. GT: Data curation, Funding acquisition, Resources, Software, Visualization, Writing – review & editing. LZ: Data curation, Funding acquisition, Investigation, Resources, Software, Visualization, Writing – review & editing.
