# Supplementary material for: Exploring deep learning radiomics for classifying osteoporotic vertebral fractures in X-ray images
Source: Front Endocrinol (Lausanne). 2024 Mar 28;15:1370838. doi: 10.3389/fendo.2024.1370838 (PMC11007145; doi:10.3389/fendo.2024.1370838)
Supplement: Supplementary file 1 [file DataSheet_1.docx]

**Supplemental Materials**

**Supplementary Table S1**. The DR, CT, MRI image acquisition parameters of the three centers.

|  |  | **Center I** | **Center Ⅱ** | **Center Ⅲ** |
| --- | --- | --- | --- | --- |
|  | Parameters | The Affiliated Taizhou People's Hospital of Nanjing Medical University | The Affiliated Hospital of Nanjing University of Chinese Medicine | Sir RunRun Hospital affiliated to Nanjing Medical University |
| **DR system information** | DR system | Digital Diagnost (Philip, Netherlands) or AXIOM ARISTOS MX (Siemens, Germany) | RAD SPEED M (Shimadzu, Japan) or Definium 6000 DR (GE Healthcare, USA) | Avisfos Vx DR (Siemens, Germany) |
|  | Tube voltage | Thoracic: 70-80 kVp  Lumbar: 80-90 kVp | Thoracic: 70-80 kVp  Lumbar: 80-90 kVp | Thoracic: 70-80 kVp  Lumbar: 80-90 kVp |
|  | Tube current | Thoracic:150-200mA  Lumbar:35-70mA | Thoracic:150-200mA  Lumbar:35-70mA | Thoracic:150-200mA  Lumbar:35-70mA |
| **CT system information** | CT system | 16-slice multi-detector CT scanner (Lightspeed Ultra, GE Healthcare, USA) or 128-slice Dual source CT (Somatom Definition, Siemens Healthcare, Germany) or 64-slice spiral CT (SOMATON sensation64, Siemens Healthcare, Germany) | 16-slice multi-detector CT scanner (Lightspeed Ultra, GE Healthcare, USA) or 256-slice Dual Source CT (Somatom Force, Siemens Healthcare, Germany) or 128-slice Dual Source CT (Somatom Definition, Siemens Healthcare, Germany) | 256-slice spiral CT (Brilliance iCT, ROYAL PHILIPS, Netherlands) or 64- slice multi-detector CT (Optima CT670, GE Healthcare, USA) |
| **CT scan parameters** | Tube voltage | 120 kVp or 120 kVp with  automated tube current modulation | 120 kVp or 120 kVp with  automated tube current modulation | 120 kVp with  automated tube current modulation |
|  | Tube current | 125 – 310 mA or 185 mA with automated tube current modulation | 118 – 320 mA or 180 mA with automated tube current modulation | 180 mA with automated tube current modulation |
| **CT image information** | Image matrix | 512×512 | 512×512 | 512×512 |
|  | layer thickness and layer interval | 1 mm, 1 mm | 1 mm, 1 mm | 1 mm, 1 mm |
| **MRI system information** | MRI system | Verio 3.0T (Siemens Healthcare, Germany) or Skyra 3.0T (Siemens Healthcare, Germany ) or Ingenia CX 3.0T (PHILIPS, Netherlands) | MAGNETOM Prisma 3.0T (Siemens Healthcare, Germany) or Achieva TX 3.0T (Philip, Netherlands) | Ingenia CX 3.0T (PHILIPS, Netherlands) or Magnetom Verio 3.0T (Siemens Healthcare, Germany) |

**Supplementary S2.** The learning rate varied with the number of iterations

$$\eta_{t}^{task-spec}=\eta_{\min}^{i}+\frac{1}{2}(\eta_{\max}^{i}-\eta_{\min}^{i})(1+\cos(\frac{T_{cur}}{T_{i}}\pi))$$

Where$\eta_{\min}^{i}=0$,$\eta_{\max}^{i}=0.01$,$T_{i}=30$represent the minimum learning rate, the maximum learning rate, and the number of iterative epochs, respectively. Because the backbone part uses pretraining parameters, to ensure the transfer effect, we have

$$T_{cur}=\frac{1}{2}T_{i}$$

The parameters of the backbone part were fine-tuned. Therefore, the learning rate of the backbone part was as follows:

$$\eta_{t}^{backbone}=\left\{\begin{matrix} \begin{matrix} 0 & & & & & & & & & & & T_{cur}\leq\frac{1}{2}T_{i} \end{matrix} \\ \begin{matrix} \eta_{\min}^{i}+\frac{1}{2}(\eta_{\max}^{i}-\eta_{\min}^{i})(1+\cos(\frac{T_{cur}}{T_{i}}\pi)) & T_{cur}>\frac{1}{2}T_{i} \end{matrix} \end{matrix}\right.$$

**Supplementary S3**

**Feature extraction**

The open-source software package Pyradiomics (http://pypi.org/project/pyradiomics/) based on Python 3.7 platform was used to extract radiomics features, including first-order features, shape features, gray level co-occurrence matrix (GLCM) features, gray level size zone matrix (GLSZM) features, gray level run length matrix (GLRLM) features, neighboring gray tone difference matrix (NGTD) features, and gray level dependence matrix (GLDM) features. A detailed description of the radiomics features extracted in this study can be found in the Pyradiomics documentation (<http://pyradiomics.readthedocs.io>). To minimize any type of bias and overfitting caused by excessive features, feature selection was performed in two steps, including intraclass correlation coefficients (ICC) and least absolute shrinkage and selection operator (LASSO) Cox regression.

The input images were resized to 64×64 using linear interpolation, and the pixel intensities were normalized to have a mean of 0 and a standard deviation of 1. Since the transfer features were extracted from the second-to-last layer of the model (AveragePooling layer), the model parameters were divided into two parts: the backbone part and the task-specific part.

**Supplementary S4**

**Feature selection & fusion**

Ten-fold cross-validation was performed based on the minimum criterion to determine the optimal λ value. Based on the model corresponding to the optimal λ value, radiomics parameters with non-zero coefficients and their weights were selected. Correlation analysis was performed on the selected features to eliminate redundancy, and parameters with a correlation coefficient greater than 0.5 were removed, resulting in a final selection of independent and stable radiomics features. Since the dimensionality of the deep transfer learning features was 2048, principal component analysis was used to reduce the dimensionality of the deep transfer learning features to ensure balance between the features, improve the generalization ability of the model, and reduce the risk of overfitting.

After selecting the radiomics features and deep learning features, Z-score normalization was applied to the fused features. The mean and standard deviation of each column feature were calculated, and each column feature was subtracted by the mean and divided by the standard deviation to transform it into a standard normal distribution. In the feature fusion stage, early fusion was performed on the radiomics features and deep learning features to form a complete feature set. Finally, the LASSO-Cox method was used to select the non-zero coefficient features and perform feature selection and dimensionality reduction on the fused features to find the optimal feature subset.


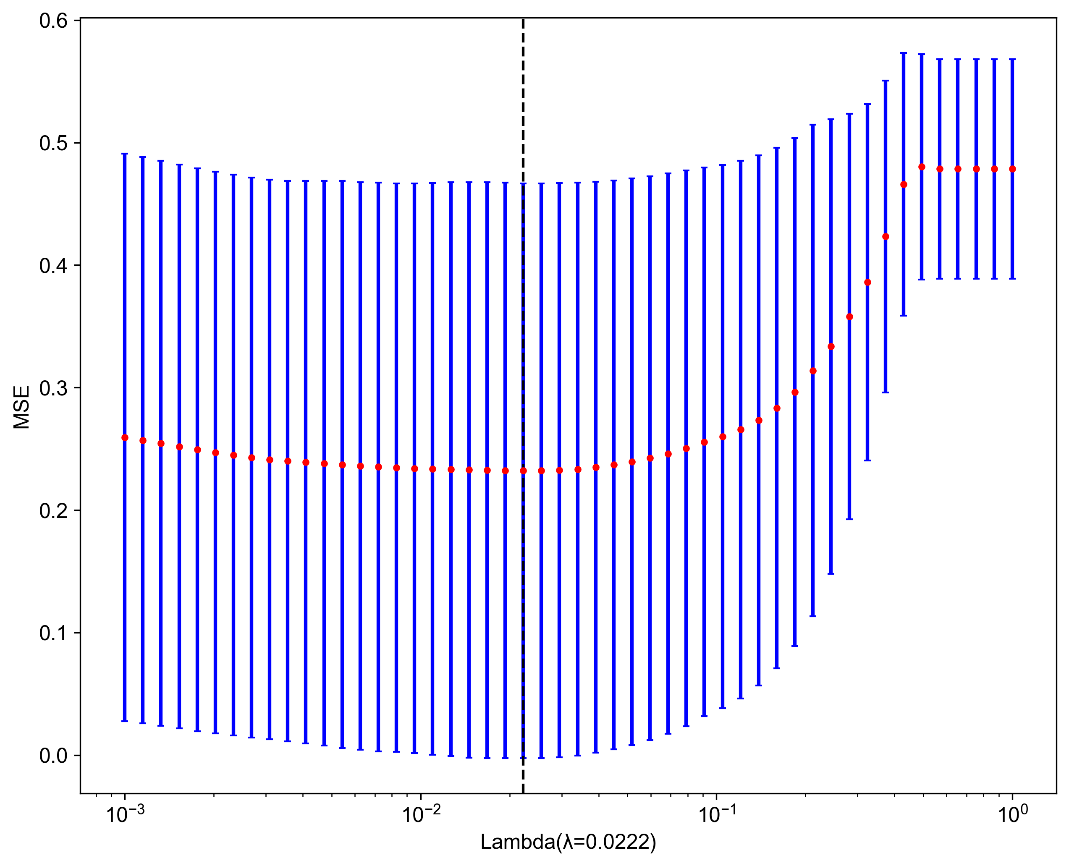

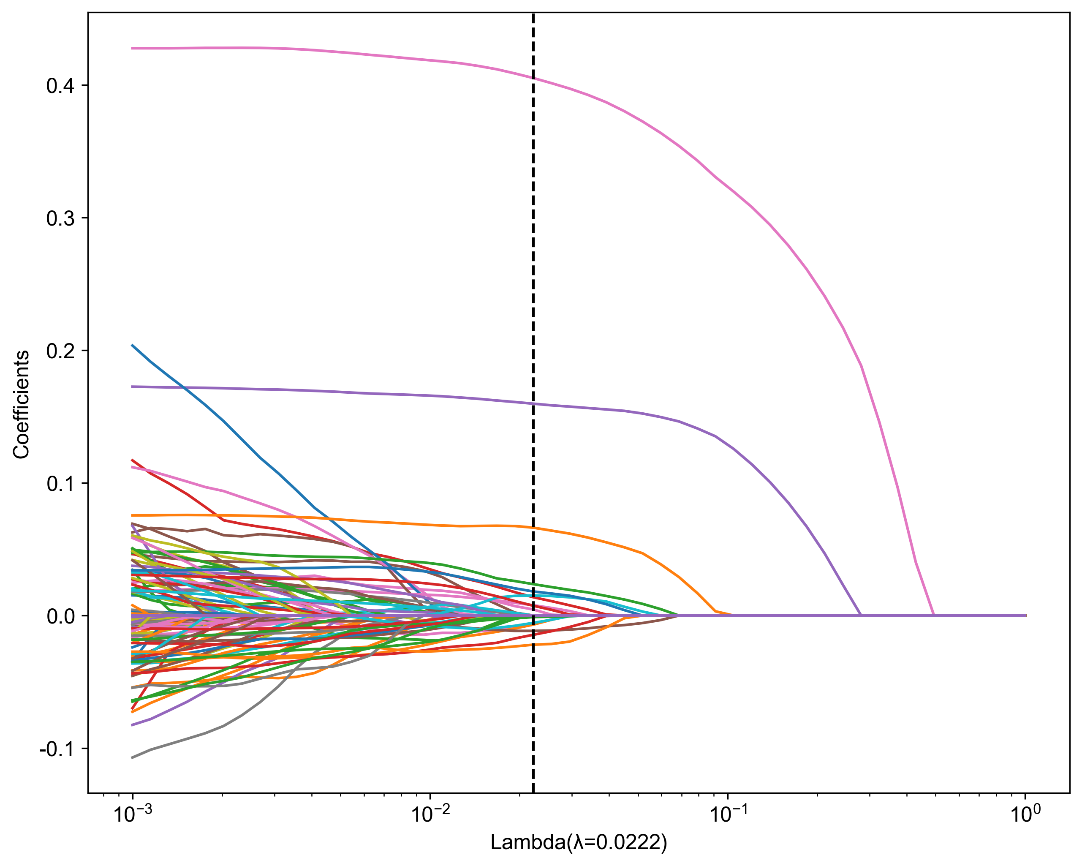


**Supplementary Figure S5** Selection of fusion features using the least absolute shrinkage method and analyzing the distribution of Deep Learning Radiomics feature importance scores from chosen features. The optimal lambda (λ) value determined was 0.0222.


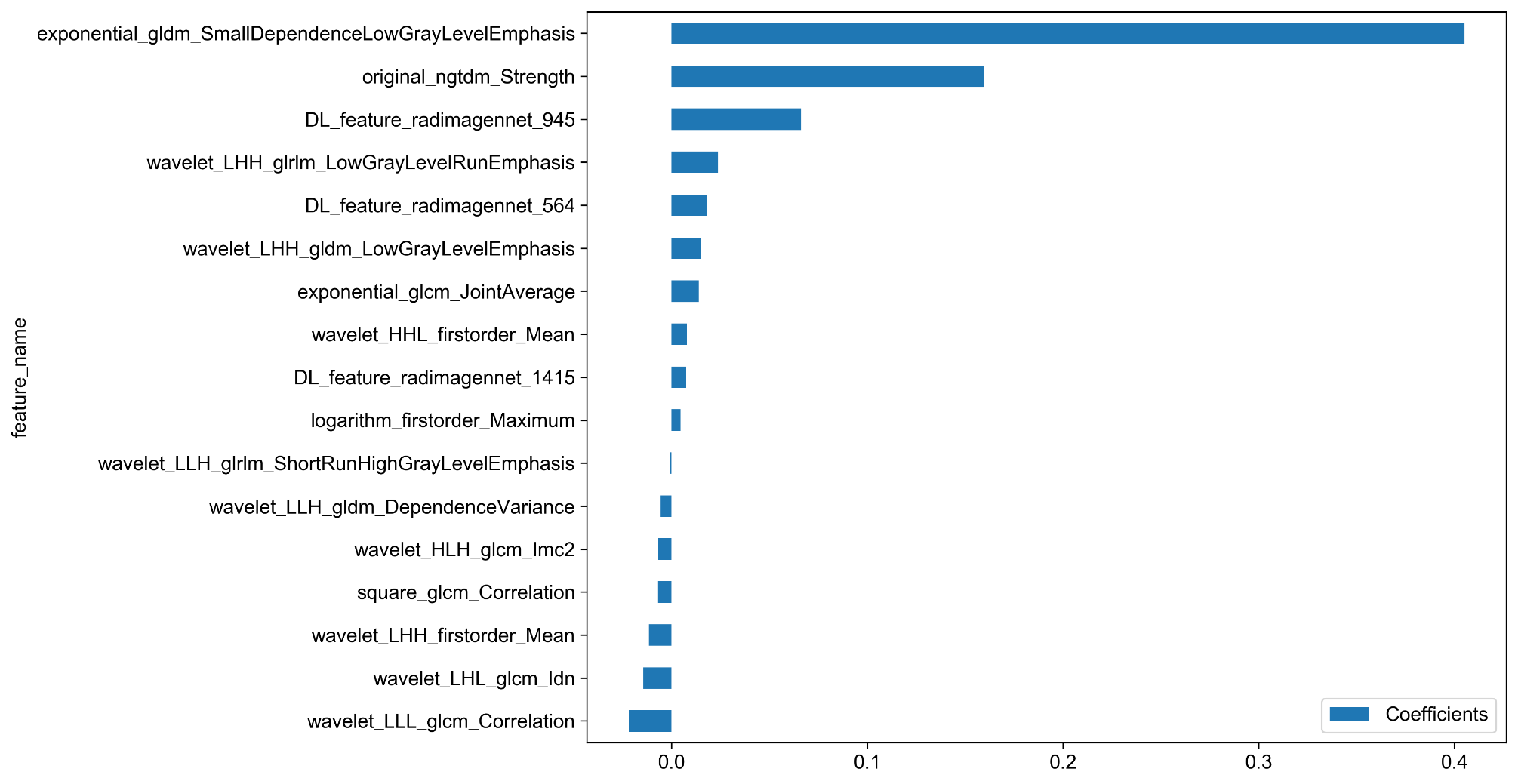


**Supplementary Figure S6** The histogram of the DTL_Radscore: the y-axis indicates the selected 17 features, and the x-axis represents the coefficient of radiomics.

**Supplementary S7**

Deep Learning Radiomics feature importance score = 0.6601123595505618 + +0.013802 * exponential_glcm_JointAverage +0.405091 * exponential_gldm_SmallDependenceLowGrayLevelEmphasis +0.004568 * logarithm_firstorder_Maximum +0.159658 * original_ngtdm_Strength -0.006867 * square_glcm_Correlation +0.007748 * wavelet_HHL_firstorder_Mean -0.006856 * wavelet_HLH_glcm_Imc2 -0.011608 * wavelet_LHH_firstorder_Mean +0.015085 * wavelet_LHH_gldm_LowGrayLevelEmphasis +0.023650 * wavelet_LHH_glrlm_LowGrayLevelRunEmphasis -0.014531 * wavelet_LHL_glcm_Idn -0.005557 * wavelet_LLH_gldm_DependenceVariance -0.001177 * wavelet_LLH_glrlm_ShortRunHighGrayLevelEmphasis -0.021935 * wavelet_LLL_glcm_Correlation +0.018095 * DL_feature_radimagennet_564 +0.066234 * DL_feature_radimagennet_945 +0.007378 * DL_feature_radimagennet_1415


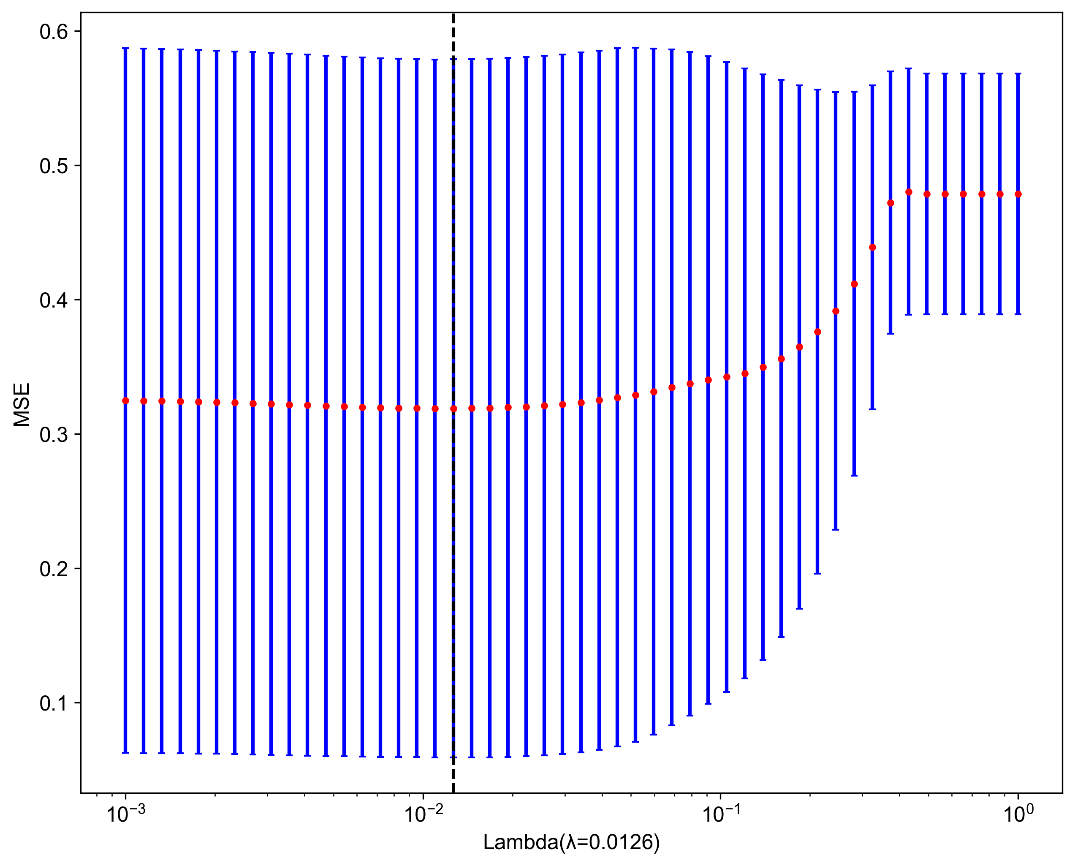

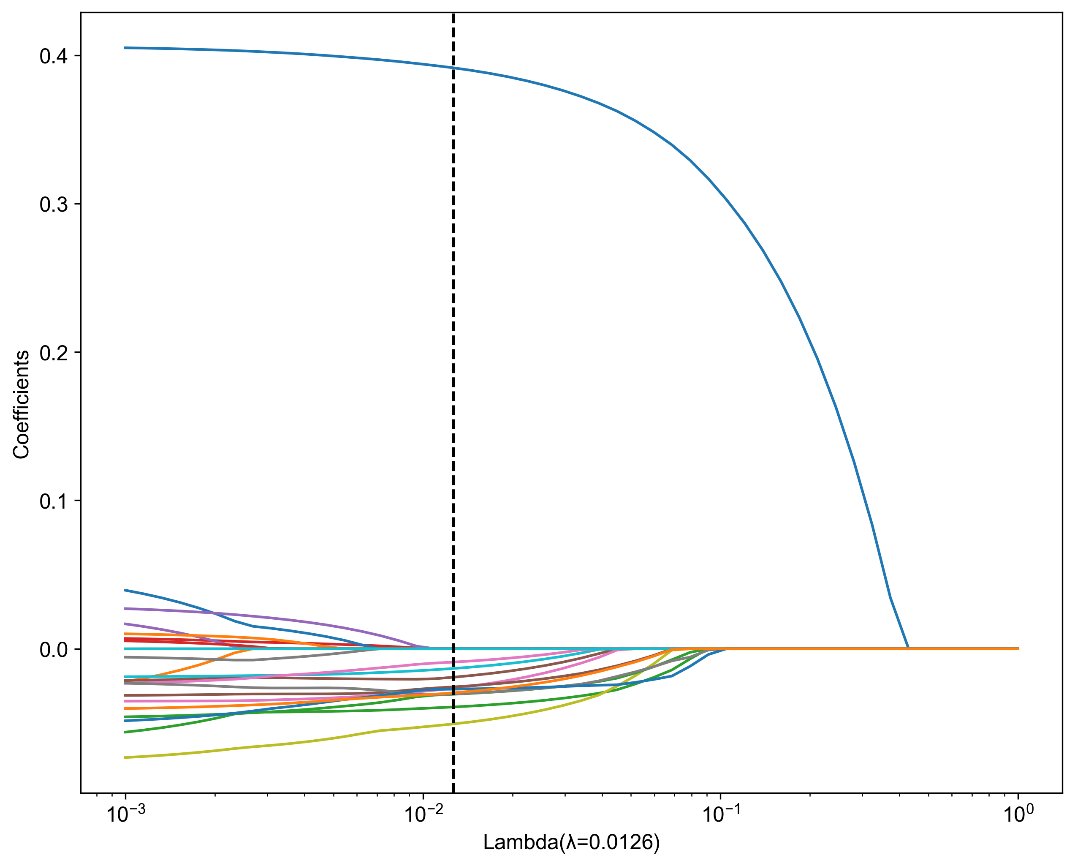


**Supplementary Figure S8** Selection of fusion features using the least absolute shrinkage method and analyzing the distribution of Deep Learning Radiomics feature importance scores from chosen features. The optimal lambda (λ) value determined was 0.0126.


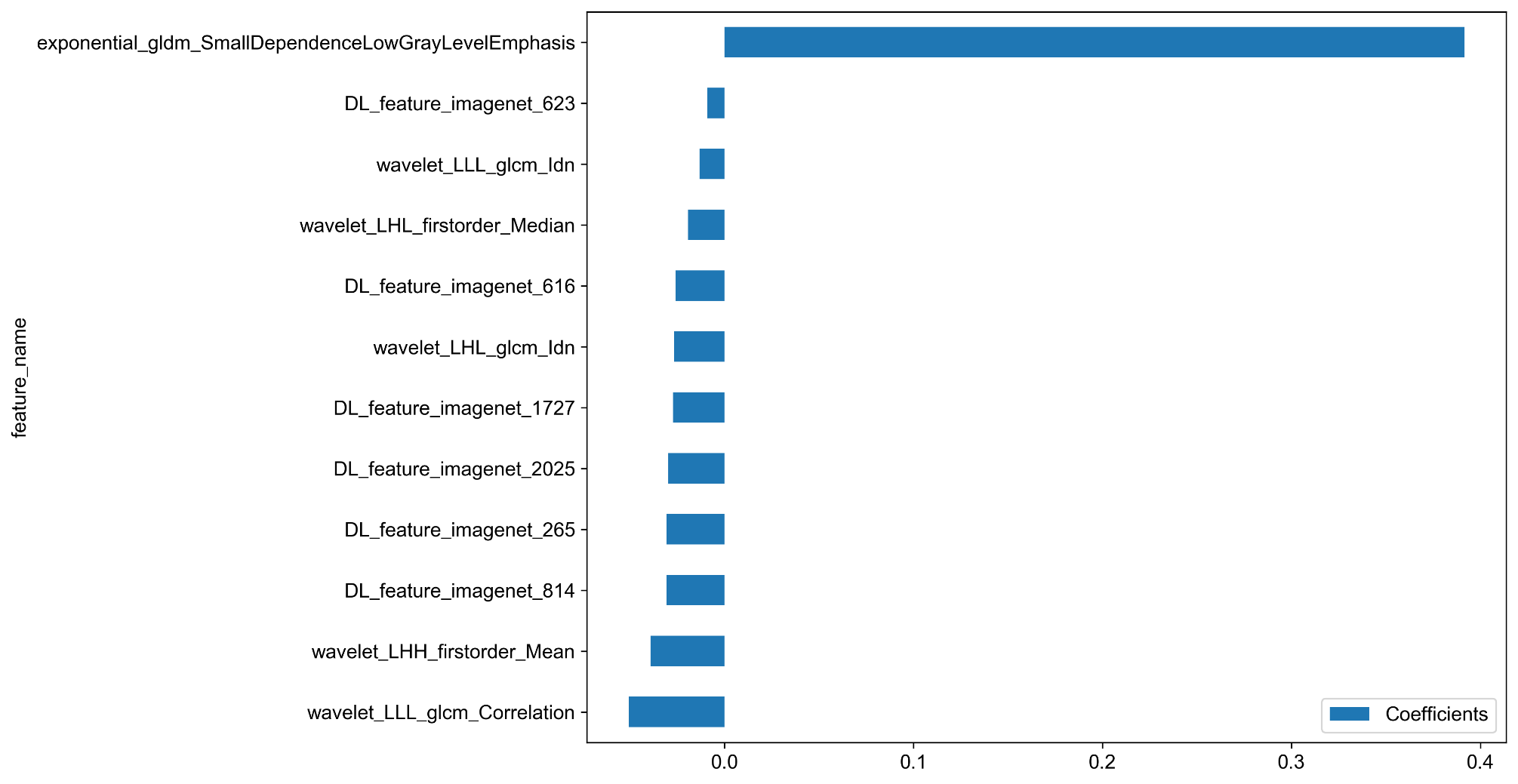


**Supplementary Figure S9** The histogram of the DTL_Radscore: the y-axis indicates the selected 12 features, and the x-axis represents the coefficient of radiomics.

**Supplementary S10**

Deep Learning Radiomics feature importance score = 0.6601123595505618 + +0.391552 * exponential_gldm_SmallDependenceLowGrayLevelEmphasis -0.039250 * wavelet_LHH_firstorder_Mean -0.019213 * wavelet_LHL_firstorder_Median -0.026621 * wavelet_LHL_glcm_Idn -0.050689 * wavelet_LLL_glcm_Correlation -0.013307 * wavelet_LLL_glcm_Idn -0.030670 * DL_feature_imagenet_265 -0.025751 * DL_feature_imagenet_616 -0.008963 * DL_feature_imagenet_623 -0.030681 * DL_feature_imagenet_814 -0.027140 * DL_feature_imagenet_1727 -0.029719 * DL_feature_imagenet_2025

**Supplementary Table S11.** Diagnostic efficiency of different models with Features Fusion in the Training Cohort, Validation Cohort and Prospective Cohort.

| **model_name** | **Accuracy** | **Macro-average AUC** | **95% CI** | **Sensitivity** | **Specificity** | **F1-score** | **RadImageNet-based task** |
| --- | --- | --- | --- | --- | --- | --- | --- |
| LR | 0.825 | 0.913 | 0.886-0.940 | 0.713 | 0.898 | 0.737 | training cohort |
| LR | 0.837 | 0.926 | 0.885-0.968 | 0.709 | 0.902 | 0.737 | Interval Validation Cohort |
| LR | 0.802 | 0.940 | 0.890-0.989 | 0.693 | 0.890 | 0.696 | External Validation Cohort |
| LR | 0.852 | 0.913 | 0.860-0.967 | 0.771 | 0.917 | 0.786 | Prospective Cohort |
| SVM | 0.882 | 0.964 | 0.947-0.981 | 0.818 | 0.928 | 0.852 | training cohort |
| SVM | 0.848 | 0.936 | 0.897-0.975 | 0.759 | 0.908 | 0.798 | Interval Validation Cohort |
| SVM | 0.856 | 0.962 | 0.923-1.000 | 0.814 | 0.912 | 0.847 | External Validation Cohort |
| SVM | 0.859 | 0.912 | 0.858-0.966 | 0.792 | 0.915 | 0.827 | Prospective Cohort |
| KNN | 0.825 | 0.947 | 0.926-0.968 | 0.733 | 0.895 | 0.766 | training cohort |
| KNN | 0.646 | 0.853 | 0.795-0.912 | 0.499 | 0.791 | 0.484 | Interval Validation Cohort |
| KNN | 0.757 | 0.875 | 0.804-0.945 | 0.581 | 0.854 | 0.583 | External Validation Cohort |
| KNN | 0.796 | 0.880 | 0.817-0.942 | 0.686 | 0.879 | 0.713 | Prospective Cohort |
| RandomForest | 0.995 | 0.999 | 0.999-1.000 | 0.989 | 0.997 | 0.992 | training cohort |
| RandomForest | 0.937 | 0.943 | 0.895-0.991 | 0.952 | 0.960 | 0.953 | Interval Validation Cohort |
| RandomForest | 0.876 | 0.891 | 0.840-0.942 | 0.839 | 0.927 | 0.866 | External Validation Cohort |
| RandomForest | 0.880 | 0.961 | 0.926-0.997 | 0.809 | 0.933 | 0.831 | Prospective Cohort |
| ExtraTrees | 0.998 | 0.999 | 0.999-1.000 | 0.999 | 0.999 | 0.999 | training cohort |
| ExtraTrees | 0.854 | 0.937 | 0.899-0.976 | 0.751 | 0.912 | 0.787 | Interval Validation Cohort |
| ExtraTrees | 0.910 | 0.964 | 0.926-1.000 | 0.906 | 0.946 | 0.907 | External Validation Cohort |
| ExtraTrees | 0.887 | 0.963 | 0.927-0.998 | 0.814 | 0.933 | 0.848 | Prospective Cohort |
| XGBoost | 0.989 | 1.000 | 0.998-1.000 | 0.985 | 0.994 | 0.987 | training cohort |
| XGBoost | 0.882 | 0.911 | 0.865-0.957 | 0.858 | 0.931 | 0.880 | Interval Validation Cohort |
| XGBoost | 0.937 | 0.950 | 0.905-0.995 | 0.952 | 0.960 | 0.953 | External Validation Cohort |
| XGBoost | 0.880 | 0.978 | 0.950-1.000 | 0.824 | 0.934 | 0.837 | Prospective Cohort |
| LightGBM | 0.928 | 0.993 | 0.985-1.000 | 0.885 | 0.958 | 0.909 | training cohort |
| LightGBM | 0.882 | 0.886 | 0.834-0.938 | 0.858 | 0.931 | 0.880 | Interval Validation Cohort |
| LightGBM | 0.937 | 0.953 | 0.910-0.997 | 0.952 | 0.960 | 0.953 | External Validation Cohort |
| LightGBM | 0.901 | 0.969 | 0.936-1.000 | 0.840 | 0.942 | 0.871 | Prospective Cohort |
| MLP | 0.856 | 0.938 | 0.915-0.960 | 0.771 | 0.915 | 0.805 | training cohort |
| MLP | 0.781 | 0.925 | 0.883-0.967 | 0.623 | 0.867 | 0.635 | Interval Validation Cohort |
| MLP | 0.811 | 0.942 | 0.893-0.990 | 0.647 | 0.890 | 0.659 | External Validation Cohort |
| MLP | 0.845 | 0.913 | 0.860-0.967 | 0.753 | 0.909 | 0.783 | Prospective Cohort |

Abbreviations: AUC, area under the curve; CI, confidence interval.

**Supplementary Table S12.** Diagnostic efficiency of different models with Features Fusion in the Training Cohort, Validation Cohort and Prospective Cohort.

| **model_name** | **Accuracy** | **Macro-average AUC** | **95% CI** | **Sensitivity** | **Specificity** | **F1-score** | **ImageNet-based task** |
| --- | --- | --- | --- | --- | --- | --- | --- |
| LR | 0.752 | 0.831 | 0.794-0.867 | 0.593 | 0.852 | 0.586 | training cohort |
| LR | 0.787 | 0.826 | 0.762-0.890 | 0.587 | 0.872 | 0.554 | Interval Validation Cohort |
| LR | 0.766 | 0.844 | 0.766-0.922 | 0.614 | 0.865 | 0.624 | External Validation Cohort |
| LR | 0.761 | 0.872 | 0.808-0.937 | 0.602 | 0.858 | 0.600 | Prospective Cohort |
| SVM | 0.807 | 0.919 | 0.893-0.944 | 0.771 | 0.882 | 0.750 | training cohort |
| SVM | 0.815 | 0.927 | 0.885-0.969 | 0.738 | 0.890 | 0.773 | Interval Validation Cohort |
| SVM | 0.766 | 0.899 | 0.836-0.963 | 0.587 | 0.857 | 0.591 | External Validation Cohort |
| SVM | 0.817 | 0.842 | 0.771-0.914 | 0.744 | 0.888 | 0.783 | Prospective Cohort |
| KNN | 0.803 | 0.928 | 0.904-0.952 | 0.700 | 0.882 | 0.732 | training cohort |
| KNN | 0.725 | 0.810 | 0.744-0.876 | 0.598 | 0.838 | 0.610 | Interval Validation Cohort |
| KNN | 0.631 | 0.661 | 0.555-0.767 | 0.462 | 0.782 | 0.447 | External Validation Cohort |
| KNN | 0.810 | 0.883 | 0.821-0.945 | 0.710 | 0.885 | 0.747 | Prospective Cohort |
| RandomForest | 0.981 | 0.999 | 0.997-1.000 | 0.965 | 0.988 | 0.975 | training cohort |
| RandomForest | 0.865 | 0.897 | 0.847-0.946 | 0.831 | 0.922 | 0.851 | Interval Validation Cohort |
| RandomForest | 0.838 | 0.888 | 0.822-0.955 | 0.828 | 0.901 | 0.852 | External Validation Cohort |
| RandomForest | 0.810 | 0.911 | 0.856-0.965 | 0.727 | 0.892 | 0.747 | Prospective Cohort |
| ExtraTrees | 0.998 | 0.999 | 0.999-1.000 | 0.999 | 0.999 | 0.999 | training cohort |
| ExtraTrees | 0.803 | 0.925 | 0.883-0.967 | 0.686 | 0.884 | 0.717 | Interval Validation Cohort |
| ExtraTrees | 0.748 | 0.875 | 0.85-0.946 | 0.601 | 0.849 | 0.619 | External Validation Cohort |
| ExtraTrees | 0.838 | 0.896 | 0.838-0.954 | 0.775 | 0.902 | 0.811 | Prospective Cohort |
| XGBoost | 0.975 | 0.999 | 0.996-1.000 | 0.968 | 0.985 | 0.974 | training cohort |
| XGBoost | 0.882 | 0.927 | 0.885-0.968 | 0.858 | 0.931 | 0.880 | Interval Validation Cohort |
| XGBoost | 0.865 | 0.935 | 0.884-0.986 | 0.901 | 0.917 | 0.901 | External Validation Cohort |
| XGBoost | 0.831 | 0.947 | 0.905-0.989 | 0.758 | 0.906 | 0.772 | Prospective Cohort |
| LightGBM | 0.870 | 0.980 | 0.978-0.993 | 0.787 | 0.992 | 0.822 | training cohort |
| LightGBM | 0.882 | 0.920 | 0.877-0.964 | 0.858 | 0.931 | 0.880 | Interval Validation Cohort |
| LightGBM | 0.865 | 0.936 | 0.885-0.987 | 0.901 | 0.917 | 0.901 | External Validation Cohort |
| LightGBM | 0.838 | 0.955 | 0.917-0.994 | 0.763 | 0.904 | 0.799 | Prospective Cohort |
| MLP | 0.786 | 0.887 | 0.856-0.917 | 0.653 | 0.871 | 0.672 | training cohort |
| MLP | 0.826 | 0.863 | 0.806-0.920 | 0.673 | 0.895 | 0.692 | Interval Validation Cohort |
| MLP | 0.775 | 0.855 | 0.780-0.931 | 0.568 | 0.864 | 0.542 | External Validation Cohort |
| MLP | 0.803 | 0.896 | 0.837-0.954 | 0.678 | 0.882 | 0.705 | Prospective Cohort |

Abbreviations: AUC, area under the curve; CI, confidence interval.

**
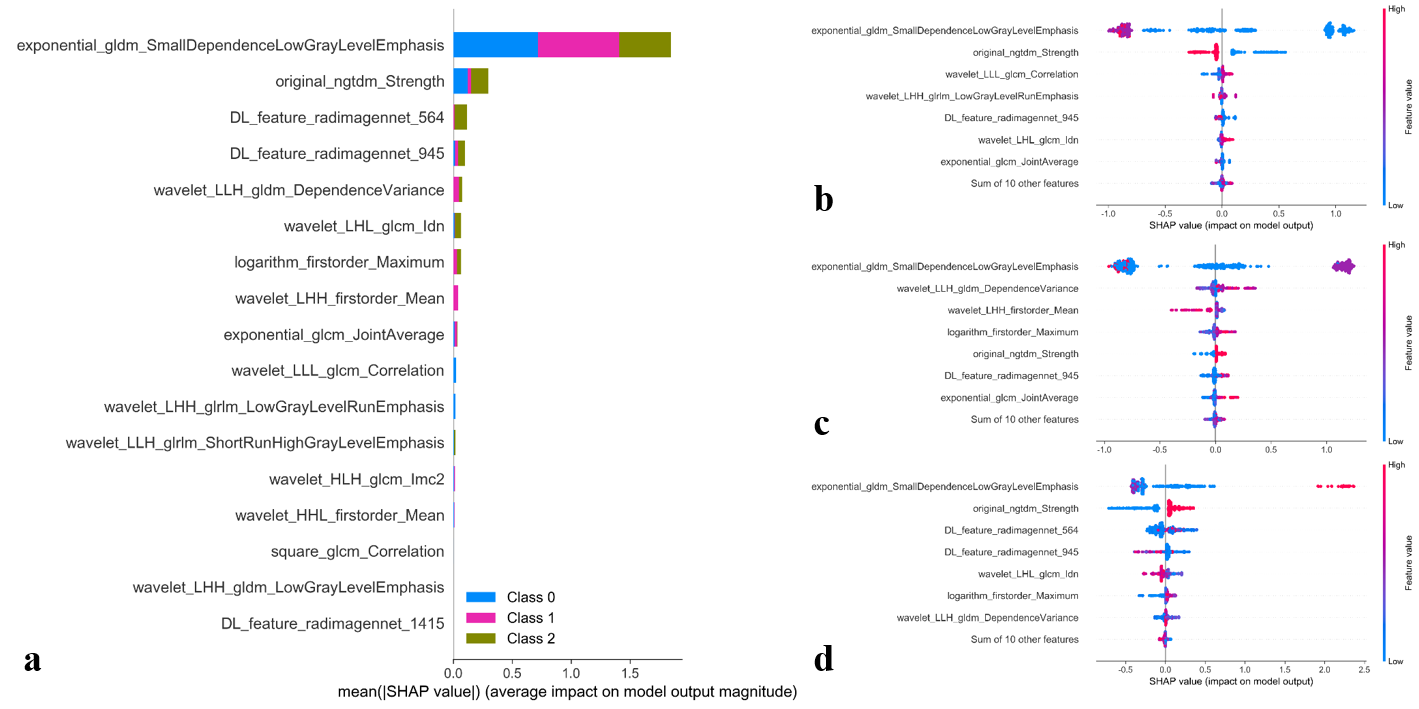
**

**Supplementary Figure S13.** Features’ contribution of the fusion model. On the y-axis are the features ordered in descending order according to their mean absolute impact on the prediction. Each distance of the dot from the axis x = 0 indicates the SHAP value for a specific feature. The farther the distance of the dot, the more effect (positive or negative) this feature had on the machine learning-deep learning model output. The color of a dot represents the original values of the features using a color bar between low (blue) and high (magenta) values. (a) The stacking bar of the mean impact of features in three classes. The blue, red, and green bars represent the Class 0, 1, 2, respectively. (b) Descending contributing features for prediction of class 0. (c) Descending contributing features for prediction of class 1. (d) Descending contributing features for prediction of class 2. SHAP, Shapley additive explanations.


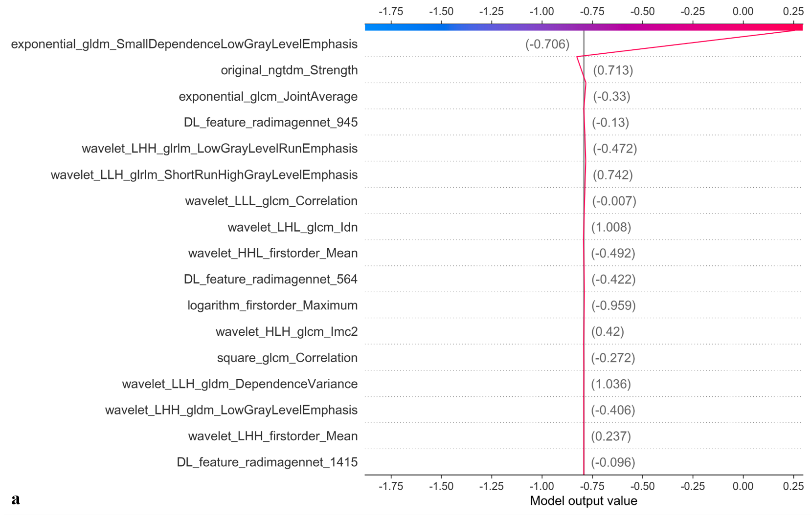

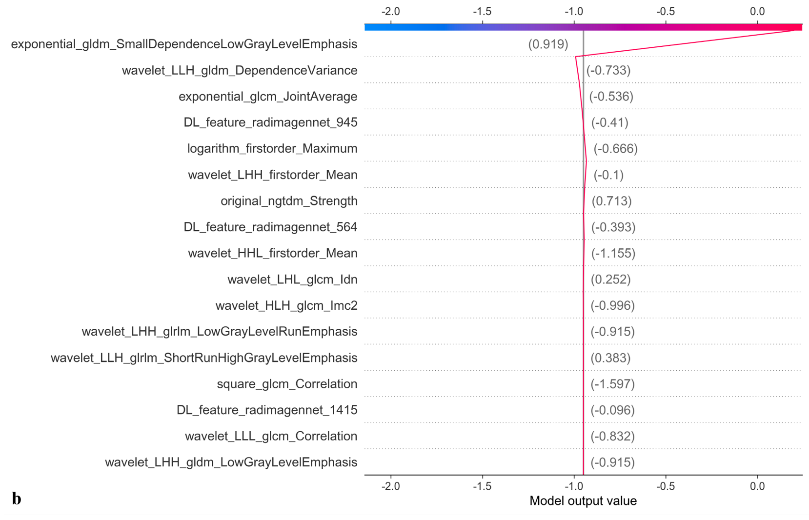

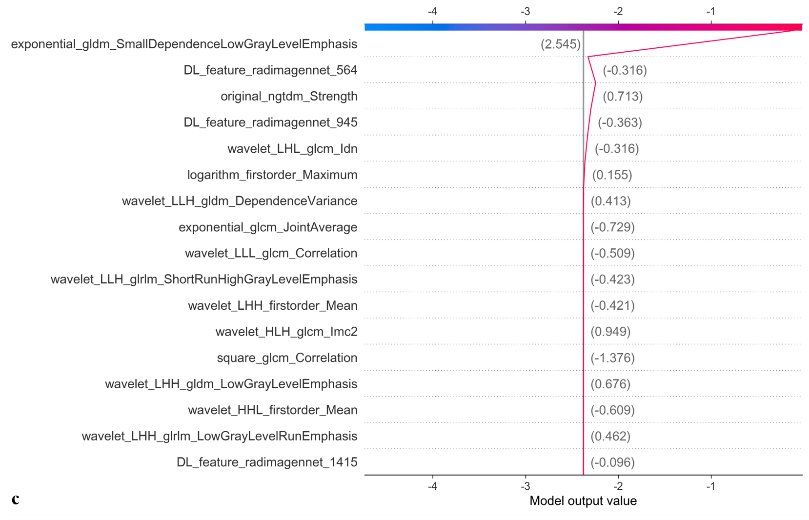


**Supplementary Figure S14.** SHAP Decision Plot: The X-axis represents the model's output, and the Y-axis represents the feature names. The gray vertical line in the middle is the baseline, and the line represents the prediction process. From bottom to top, the baseline values (A: Class 0, B: Class 1, C: Class 2) are -0.791, -0.949, -2.375, respectively. Through the interaction of positive and negative feature values, the final model output values are 0.254, 0.198, -0.108. These decision plots help explain the contribution of different features to the model and the prediction process.
